# Dorsal Root Injury—A Model for Exploring Pathophysiology and Therapeutic Strategies in Spinal Cord Injury

**DOI:** 10.3390/cells10092185

**Published:** 2021-08-25

**Authors:** Håkan Aldskogius, Elena N. Kozlova

**Affiliations:** Laboratory of Regenertive Neurobiology, Biomedical Center, Department of Neuroscience, Uppsala University, 75124 Uppsala, Sweden; elena.kozlova@neuro.uu.se

**Keywords:** nerve regeneration, nerve degeneration, sensory neuron, astrocyte, microglia, gene regulation, trophic factor, transplantation

## Abstract

Unraveling the cellular and molecular mechanisms of spinal cord injury is fundamental for our possibility to develop successful therapeutic approaches. These approaches need to address the issues of the emergence of a non-permissive environment for axonal growth in the spinal cord, in combination with a failure of injured neurons to mount an effective regeneration program. Experimental in vivo models are of critical importance for exploring the potential clinical relevance of mechanistic findings and therapeutic innovations. However, the highly complex organization of the spinal cord, comprising multiple types of neurons, which form local neural networks, as well as short and long-ranging ascending or descending pathways, complicates detailed dissection of mechanistic processes, as well as identification/verification of therapeutic targets. Inducing different types of dorsal root injury at specific proximo-distal locations provide opportunities to distinguish key components underlying spinal cord regeneration failure. Crushing or cutting the dorsal root allows detailed analysis of the regeneration program of the sensory neurons, as well as of the glial response at the dorsal root-spinal cord interface without direct trauma to the spinal cord. At the same time, a lesion at this interface creates a localized injury of the spinal cord itself, but with an initial neuronal injury affecting only the axons of dorsal root ganglion neurons, and still a glial cell response closely resembling the one seen after direct spinal cord injury. In this review, we provide examples of previous research on dorsal root injury models and how these models can help future exploration of mechanisms and potential therapies for spinal cord injury repair.

## 1. Introduction

Dorsal root ganglion (DRG) neurons are pseudounipolar cells with a single process, bifurcating in a peripherally and a centrally directed branch. The latter initially shares the typical features of peripheral nerve tissue with Schwann cells and basement lamina. Just outside the spinal cord, at the dorsal root transitional/entry zone (DRTZ/DREZ), dorsal root axons encounter central glia and course through channels between astrocytes [1,2,3]. Myelinated axons that enter the spinal cord are myelinated by Schwann cells peripherally and by oligodendrocytes centrally, forming a so-called transitional node at the DREZ [4]. Since myelination in the central nervous system (CNS) is triggered at axonal diameters smaller than in the peripheral nervous system (PNS) [5], large non-myelinated sensory axons in the PNS are likely to become myelinated after their entry into the spinal cord [6]. DRG harbors a range of neuronal subtypes, reflecting multiple tactile, proprioceptive and visceral, and nociceptive sensory functions [7,8,9,10,11]. Thus, dorsal root injury models allow the analysis of regenerative potential related to structural and/or functional properties of the affected neurons.

Disorders that damage dorsal roots include compression, (e.g., from herniated disc), infections, autoimmune mechanisms, metabolic disorder, neurotoxic agents, and trauma. Clinically, the most important type of traumatic injury of the dorsal root is spinal root avulsion, a forceful, longitudinal traction of spinal nerve roots, interrupting their connections with the spinal cord. This condition may occur during complicated delivery, from a fall from a high height, or in a traffic accident, and most commonly affects spinal nerve roots innervating the upper extremity, i.e., brachial plexus avulsion.

Root avulsion injuries in experimental animals are typically performed by a surgical approach after exposure of the spinal cord and appropriate root entry regions to achieve standardized and reproducible lesions. However, in clinical root avulsion injuries, the forces that pull the roots from the spinal cord are usually exercised from outside the vertebral canal. The lesion is variable in location and extent of injury, and with possible differences in, for example, the consequences for the regional vascular supply. In terms of location, clinical avulsion injuries can occur distal (postganglionic injury) or proximal (preganglionic injury) to the dorsal root ganglia [12]. The postganglionic avulsion injury has the character of a peripheral nerve injury with the possibility for functional recovery, whereas a preganglionic avulsion injury is, in principle, a longitudinal injury of the spinal cord at the entry of spinal nerve roots, and thus provides similar therapeutic challenges as other spinal cord injuries [13].

Spinal root avulsion injuries typically affect ventral and well as dorsal roots, causing paralysis of denervated muscles, loss of sensory and autonomic function, and, most often, neuropathic pain. Cauda equine syndrome, which encompasses avulsion of lumbosacral roots, also leads to weakness or paralysis of muscles controlling micturition, defecation, and sexual functions. Surgical treatment of spinal root avulsion offers a possibility for partial functional restoration of motor function by reinnervation of proximally located muscles, but clinically efficient treatment for restoring sensory function is thus far not available [13,14]. Thus, dorsal root avulsion exhibits therapeutic challenges specific for this condition but also features clearly relevant for other forms of spinal cord injury. Mechanisms that are implicated in spinal cord pathophysiology and repair are covered in numerous recent reviews, e.g., reference [15].

Here, we discuss injury (axotomy) of dorsal roots as an attractive experimental model to address key aspects of spinal cord injury repair in vivo. Already Ramon y Cajal made the conclusion from his experiment that whereas injury to the peripheral branch of dorsal root ganglia is followed by axonal regeneration and reconnection with peripheral targets, injury to the central branch results in abortive axonal growth [16]. A detailed description of the different dorsal root injury approaches, their advantages and disadvantages were provided previously [17]. Restoration of functions that are lost following dorsal root injury requires that injured dorsal root axons are able to extend axons through a non-permissive environment, which is rapidly building up at the DREZ. In addition, the regenerative response of the central process of DRG neurons is markedly weaker than what follows after an injury to their peripheral process. These two factors contribute to the failure of injured dorsal root axons to re-enter the spinal cord in a similar way a non-permissive environment and attenuated growth response obstruct functional regeneration in the injured spinal cord.

Thus, axotomy (crushing or cutting) of the dorsal root in its peripheral compartment allows investigation of how axonal extension can be promoted, first along with Schwann cells, and thereafter when encountering central glia, which reacts to the injury at a distance, i.e., without damage to the spinal cord itself. On the other hand, the most centrally located dorsal root injury, dorsal root avulsion, will damage the DREZ, including its central glial component, and thereby constitute a true CNS injury, but isolated to the dorsal root axons and their immediate surroundings [13]. In this brief review, we describe how DREZ becomes a barrier for axonal ingrowth, give examples of previous and current research exploring how to overcome this barrier in the adult nervous system, and how future spinal cord injury research can benefit from using dorsal root injury models.

## 2. Development of the DREZ—From Entry to No-Entry

During development, dorsal root sensory axons enter the spinal cord in temporally successive waves [18]. A transient population of neural crest-derived cells, boundary cap cells, located at spinal cord entry/exit points [19], allow sensory axons to penetrate the spinal cord [20,21] while preventing central glia [22,23] and motor neurons [24,25] from migrating peripherally. Friedrich’s ataxia is a rare, recessive autosomal neurological disorder causing severe impairment in muscle coordination. It is characterized pathologically by abnormalities of myelinated axons in the peripheral nervous system, and spinal cord dorsal column, and of extensive peripheral migration of astrocytes into the dorsal roots [26].

**Figure 1:**
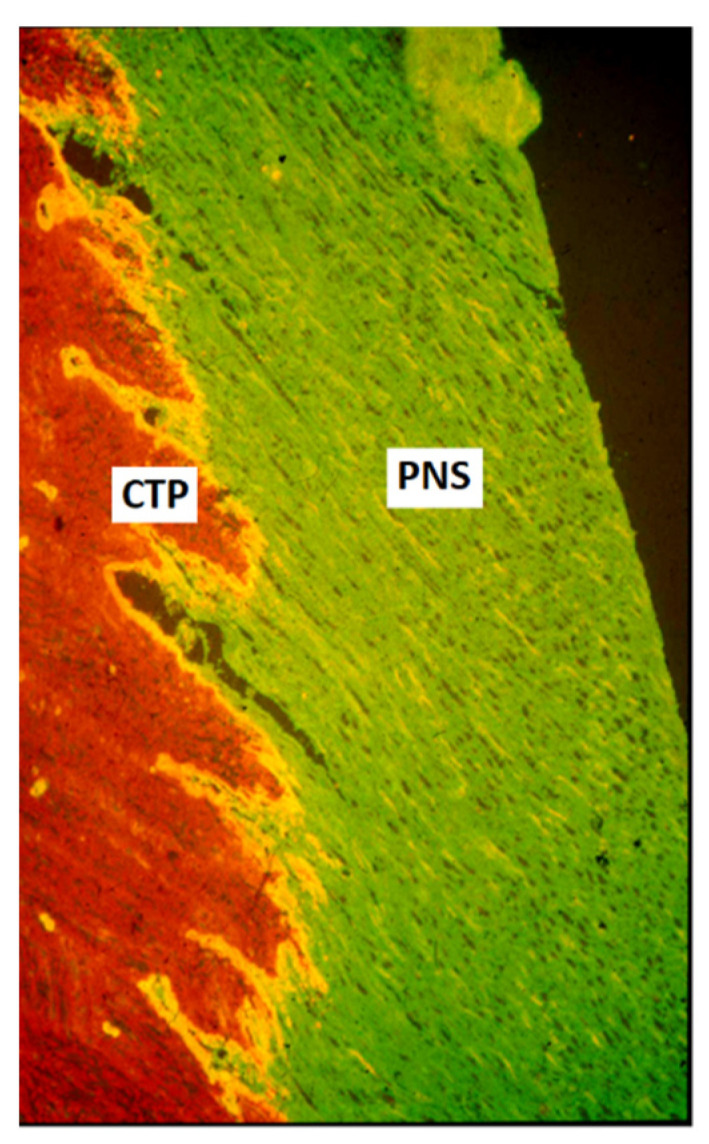
Intact, the lumbar dorsal root of the adult rat. Horizontal section through several dorsal rootlets, immunostained with antibodies to laminin (green) and glial fibrillary acidic protein (GFAP; red). The dorsal root transitional zone is formed by cone-shaped peripheral projections of central nervous tissue, so-called central tissue projections (CTP; red), which interdigitate with the peripheral nervous compartment of the root (green). ×200. Reproduced with permission from Aldskogius, H., Kozlova, E.N., Brain Research Reviews; published by Elsevier, 2002 [35].

The regulated sensory axon ingrowth appears to be dependent on semaphorin [25,27] and netrin [28,29] signaling. During the late fetal or early postnatal period, the boundary between PNS and CNS becomes a barrier for axonal entry [2,3,30,31]. This barrier is located some distance outside the spinal cord surface, and the central part, containing astrocytes and oligodendrocytes forms, at most levels, dome-shaped central tissue projections (Figure 1). The emergence of this barrier correlates with the deposition of extracellular matrix molecules, such as cytotactin/tenascin and chondroitin 6-sulfate-containing proteoglycans (CPSG) [32,33,34]. The PNS-CNS interface is easily visualized, e.g., by immunolabeling with anti-laminin antibodies for the PNS and anti-glial fibrillary acidic protein for the CNS compartment (Figure 1).

## 3. Overcoming the Limited Regenerative Competence after Dorsal Root Injury

An appropriate cell body response, comprising a set of so-called regeneration associated genes (RAGs) [36], combined with the emergence of growth supportive axon-glial interactions at the injury site and during axonal elongation, are fundamental for successful nerve regeneration. Axotomy of dorsal root axons results in a much weaker regenerative response by the sensory neurons than what follows after injury to their peripheral branch. Rodent regenerating dorsal root axons grow with a rate of ca 1 mm/24 h compared to ca 3 mm/24 h for injured peripheral nerve axons [37] and fail to display the molecular modifications that is typical after peripheral nerve injury [38,39,40]. Furthermore, injured dorsal root axons have limited ability to grow for long distances even in a peripheral nerve environment [41,42] unless actions to enhance their intrinsic regenerative potential are taken.

Although DRG is composed of phenotypically diverse neurons, they appear to undergo a uniform transcriptional program after peripheral nerve injury, induced by cyclic AMP-dependent transcription factor (ATF-3) [43]. Whether the attenuated regenerative response after dorsal root injury is also mediated by a uniform transcriptional program is unknown. Following dorsal root injury, a neuronal size-related differential expression was shown for mRNAs of the guidance molecules semaphorins and their receptors (neuropilins), as well as for vascular endothelial growth factor (VEGF) [44], suggesting possible differences in the long-term regenerative response to axotomy of central sensory axons.

Interventions, e.g., the use of growth factors or cell-based approaches to promote regeneration of dorsal root axons, are likely to result in differential effects depending on neuronal subtype-specific growth factor receptor pattern and cell-matrix interactions signaling systems (see Section 3.3).

### 3.1. The Preconditioning Paradigm

The potential of DRG neurons to mount an efficient regenerative response after dorsal root injury is shown by a so-called conditioning lesion, i.e., an injury to the peripheral sensory axons. This intervention, which is typically performed one to two weeks prior to injury to the central processes of the same sensory neurons induces RAGs in DRG neurons and stimulates growth supportive interactions between dorsal root axons and associated non-neuronal cells, thereby promoting elongation of injured dorsal root axons [45]. The conditioning lesion paradigm has made a significant contribution to our understanding of regeneration failure after injury to primary sensory axons.

A conditioning peripheral nerve lesion by itself may allow spinal cord entry of a limited number of injured dorsal root axons [46], which appear to originate from specific DRG subpopulations [47]. An effect of a conditioning injury is also seen on axonal growth after an injury to the central sensory processes in the dorsal column [48], even when the peripheral injury is performed after the spinal cord lesion [49,50]. Since the conditioning lesion approach is not suitable for clinical application, a range of alternative strategies have been adopted to achieve spinal cord ingrowth from injured dorsal root axons. Here, we discuss a few of the most promising of these strategies.

### 3.2. Axonal Protein Synthesis and Axon Regeneration

Although much of the regeneration-associated neuronal changes occur in the cell bodies of the affected neurons, the importance of a local regeneration supporting metabolic system, proposed already long ago [51,52], is now well established [53,54,55,56,57]. Such metabolic machinery is highly relevant for the repair of human nerve injury since the distance between the site of axotomy and the affected nerve cell body is often considerable. Axonally transported ribosomes and mRNA, together with axonal uptake of ribosomes and mRNA from Schwann cell-derived exosomes, enables initiation of axonal elongation without the early support from the nerve cell body. The starting point and time course of this process may be critical for the possibility of injured axons to negotiate a reactive CNS environment. Transfer of microvesicles from Schwann-like cells has shown promising beneficial effects on axonal growth [58]. To speed up the initiation of the local axonal regenerative machinery might offer a window of opportunity for spinal cord ingrowth of injured dorsal root axons before the fully non-permissive barrier at the DREZ is formed.

### 3.3. Growth Factors for Promoting Regeneration of Injured Dorsal Root Axons

Extracellular [59] or gene transfer mediated [60] administration of selected growth factors has led to a marked improvement in the regenerative capacity of injured dorsal root axons. The most striking effects have been shown with intrathecal delivery of neurotrophin (NT)3, or glial cell line-derived neurotrophic factor (GDNF) [61,62], or systemic administration of Artemin [63,64,65], a member of the GDNF family. The combined release of peptide mimetics of ciliary neurotrophic factor (CNTF) and GDNF from mesoporous silica nanoparticles implanted on the avulsed DREZ supported partial sensory axon ingrowth [66]. Since these effects are mediated after binding to specific receptors, which are expressed by subpopulations of DRG neurons, a combination of multiple growth factors would be required to achieve the broadest possible impact. Treatment with platelet-rich plasma, a likely source of an abundance of growth factors, was shown to partially restore sensorimotor function after dorsal root injury and may meet the requirement for multiple growth factor administration [67].

Growth factor-mediated growth of dorsal root axons appears to act on intracellular pathways, which at least in part share those implicated in the conditioning lesion response [68], although simultaneous effects on non-neuronal cells at the DREZ may also occur [69]. Importantly, there appears to be a limited time window for neurotrophin-mediated entry of regenerating dorsal root axons since just a short delay of treatment fails to support spinal cord ingrowth [70]. This emphasizes the desire to initiate growth supportive therapy as soon as possible. Dorsal root injury is quite favorable for addressing this aspect.

### 3.4. Modification of the mTOR Pathway

PTEN (phosphatase and tensin homolog deleted on chromosome 10) has emerged as promising target to recover the intrinsic growth capacity of adult neurons. PTEN inhibits phosphoinositide 3-kinase (PI3-K)/Akt/mTOR pathway, which is critical for sustained axonal elongation. Activation of PTEN also appears to contribute to myelin-associated growth inhibition [71]. Blocking PTEN expression by gene deletion, RNA silencing, or pharmacological inhibitors significantly promotes sustained regenerative growth in vivo by CNS [72,73,74,75,76] and PNS [77,78] neurons without long-term adverse effects [79]. Treatment with a retinoic acid receptor β (RARβ) agonist was shown to modify PTEN activity, promote the growth of sensory axons, counteract the growth repelling environment at the DREZ, and support sensorimotor recovery after spinal root avulsion [80]. Further studies revealed that retinoic acid synthesis by NG2 glia is an important mechanism in providing a growth permissive CNS environment [81].

### 3.5. Targeting Microtubules

To allow elongation of regenerating axons, microtubules behind the leading edge need to be stabilized [82]. Studies in non-mammalian species even demonstrated efficient microtubule-based axon regeneration without the formation of growth cones [83]. Treatment with the microtubule-stabilizing agent taxol promoted axon regeneration in the mammalian spinal cord [84]. Furthermore, inhibition of kinesin-5, a motor protein that acts as a “brake” on microtubule advancement, promotes sensory neurite extension on a growth inhibitory substrate in vitro [85]. Down-regulation of fidgetin, a microtubule severing molecule, mobilizes an increased mass of labile microtubules, associated with the growth of dorsal root axons into the spinal cord, although evidence of functional repair was not observed [86,87]. Ingrowth of injured dorsal root axons was also shown by daily stimulation for 12 weeks of DRG neurons expressing the receptor hM3Dq, a so-called designer receptor exclusively activated by designer drugs (DREADDs), with its ligand clozapine-N-oxide (CNO), in combination with proteoglycan degrading enzyme treatment [88].

## 4. Regulating Gene Expression after Axon Injury

The mechanisms for induction and maintenance of a neuronal regeneration program, including its regulation at the gene level, were extensively reviewed [36]. From having previously focused on and clarified the expression of regeneration-associated genes, studies in recent years have begun to unravel how these genes are regulated by non-coding RNAs, and by epigenetic DNA and histone modifications. Non-coding RNAs have distinct roles in transcriptional and posttranscriptional processes and cooperate in a regulatory network for gene expression [36]. Non-coding RNAs which have been implicated in the regulation of peripheral nerve regeneration include long non-coding RNA (lncRNA) [89], micro-RNA (miRNA) [90], and circular RNA (circRNA) [91].

Following spinal cord injury, there are marked changes in the expression of non-coding RNAs, which appear to be associated with the pathophysiological events and the subsequent repair mechanisms [92]. MiR-155 resulted in improved regeneration and functional recovery after spinal cord dorsal column injury, effects that appear to be mediated through the reduced inflammatory response as well as induction of regeneration-associated genes [93]. miRNA-26a primed mesenchymal stem cells were shown to promote spinal cord repair through the mTOR pathway [94], and miR-20a and miR 155-5p promotes the growth of injured sensory axons in the spinal cord dorsal column via the cAMP/PKA [95] and PDZ-RhoGEF/RhoA/GAP43 [96] pathways, respectively. Non-coding RNAs have also been implicated in neuropathic pain [97,98], a common, severely disabling sequelae of spinal root or spinal cord lesion.

Epigenetic modification has recently emerged as a significant factor influencing the outcome after neural injury [99]. Successful peripheral nerve regeneration requires active DNA demethylation [100]. Injury of peripheral but not central sensory axons results in increased chromatin accessibility and histone acetylation. These epigenetic changes are accompanied by the induction or regeneration-associated gene expression [101]. Along the same line, DNA methylation of sequences of the Myc proto-oncogene preceded the up-regulation of a set of RAGs after a conditioning lesion of sensory neurons [102]. Interestingly, environmental enrichment prior to a spinal cord injury was shown to promote functional recovery, mediated by Creb-binding protein histone acetylation [103]. By combining environmental enrichment with a preconditioning lesion, a further enhancement of the growth capacity of injured spinal cord sensory axons has been achieved [104]. Further exploration of these findings, e.g., in dorsal root injury models, may help to develop combinatorial therapies based on interventions with bioactive agents and rehabilitative measures [105].

## 5. Growth Inhibition at the DREZ

### 5.1. Manipulating Growth Inhibitory Factors

**Figure 2:**
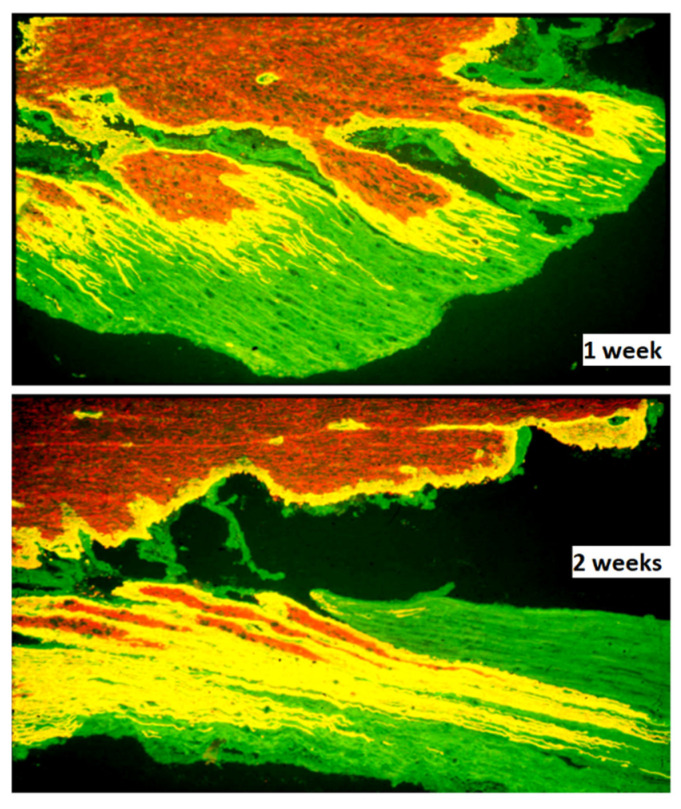
Lumbar dorsal roots following ipsilateral dorsal root injury. Horizontal sections, immunostained with antibodies to laminin (green) and GFAP (red). The extensive outgrowth of astroglial processes (red and yellow) into the peripheral compartment is shown at one week (above) and two weeks (below) following injury. ×200. Reproduced with permission from Aldskogius, H., Kozlova, E.N., Brain Research Reviews; published by Elsevier, 2002 [35].

Microglia and astrocytes play critical roles in the pathophysiology and repair after spinal cord injury and are, therefore, potential targets for therapy [106]. Reactive CNS glia creates an efficient barrier for spinal cord ingrowth of injured axons at the DREZ. Astrocytes at the DREZ and along the sensory pathways in the spinal cord proliferate [107,108], and undergo marked hypertrophy, as evidenced, for example, by the peripheral extension of long processes [35,109] (Figure 2). The growth-inhibitory influence at the DREZ is considered to be formed mainly by the deposition of proteoglycans [31,32,33] and the presence of myelin-associated growth inhibitors [110] Up-regulation of astrocyte expressed calcium-binding protein S100A4/Mts1 [111], shown to be a growth inhibitor in vitro [112,113] and in vivo after implantation of bNCSCs to the avulsed spinal cord [114], may also contribute.

Previous studies reported that a conditioning lesion combined with enzymatic degradation of chondroitinase sulfate proteoglycans with chondroitinase ABC [115], their removal through gene deletion [116], blocking Nogo-receptor function [117,118], or integrin activation [119] allow regeneration of injured dorsal root axons into the spinal cord. However, recent experiments indicate that a conditioning lesion in combination with proteoglycan degradation or blocking myelin-associated inhibitors is not sufficient in this context, and that specific growth factor stimulation, in this specific case with GDNF, is required for sensory axon entry and functional recovery [120]. This finding indicates that the mechanisms which prevent spinal cord ingrowth by injured dorsal root axons are still far from clear and need further investigations.

The limited effect on sensory axon spinal cord ingrowth by pharmacological interventions targeting the DREZ may be related to the observation that regenerating sensory axons form synapse-like contacts with non-neuronal cells, possibly NG2-positive glia, rather than traversing the PNS-CNS boundary and enter the spinal cord [121,122,123,124]. This process resembles the appearance of synapse-like connections between injured sensory axons in the dorsal column and NG2 positive glia [125]. Thus, NG2+ glia may provide cues for establishing contacts with regenerating dorsal root axons, perhaps originating from specific sensory neurons. To identify these cues in detail and apply counteracting interventions may contribute to functional dorsal root axon regeneration.

### 5.2. Clearance of Axonal and Myelin Debris

During Wallerian degeneration of peripheral nerves, Schwann cells in cooperation with fibroblasts and immune cells, clear disintegrating axons and myelin in preparation for a growth permissive pathway for axon regeneration. In contrast, myelin debris arising from Wallerian degeneration in the central nervous system remains for long periods of time [126,127]. In humans, residues of degenerating myelin have been observed decades after spinal cord injury. These residues and the associated cellular reactions appear to hamper axonal growth. To speed up myelin clearance in the injured spinal cord is thus a relevant objective for promoting spinal cord injury repair. This issue can be favorably explored after dorsal root injury, which allows examination of myelin degradation and elimination in the spinal cord dorsal column without direct damage to the spinal cord, leaving its vascular supply and tissue architecture essentially unaffected.

The long-term lingering of myelin debris after spinal cord injury is probably the result of an insufficient activation of the macrophage/microglial program required for rapid and complete myelin degradation [128,129,130]. Toll-like receptor signaling mediates myelin phagocytosis [131,132], whereas beta-amyloid precursor clearing enzyme (BACE)1 [133,134], and signal regulatory protein-α (SIRPα) [135] delays this process. These findings provide options for approaches for rapid elimination of myelin debris and, hence, possibly minimize one obstacle for sensory axon extension across the DREZ.

Small rodents have dominated experimental studies on spinal cord injury for decades, but, as often recognized, translation from these studies to humans is frequently difficult. Early studies of Wallerian degeneration in the spinal cord or animals with considerably larger myelinated axons than what is the case in the small rodents have shown the emergence and long-term presence of abnormal profiles, such as the “myelinoclasts” in monkeys [136]. Similar profiles have also been observed in the cat [137,138] but not in the rat [126,127]. These structures are formed of layers of myelin wrapped around non-neuronal cells, most likely microglia, which subsequently appear to undergo degeneration and become phagocytosed by other populations of microglia [138]. The mechanisms underlying these distinct differences in how Wallerian degeneration proceeds in larger organisms compared to rats or mice may be important to consider in translational research on spinal cord injury repair. Further analysis also needs to consider the presence of microglia/macrophage subpopulations and their properties in response to Wallerian degeneration in the spinal cord. Recent studies using single-cell RNA analysis have shown the existence of multiple macrophage populations in degenerating peripheral nerve [139] and a marked heterogeneity of microglia in neural disorders [140].

## 6. Secondary Neurodegeneration in the Spinal Cord

Dorsal root avulsion has consequences not only for spinal cord glia and immune cells but also results in degeneration of postsynaptic neurons in the dorsal as well as ventral horn [141]. Loss of neurons after spinal cord injury may be a consequence of the vascular and immune pathophysiology and thus becomes an important part of the secondary injury area [141,142]. In addition, neurons may be lost at a significant distance from both the primary and secondary lesion area as a result of diminished input to postsynaptic neurons (anterograde transneuronal degeneration).

Conversely, there is a loss of previously existing postsynaptic targets, causing presynaptic neurons to degenerate (retrograde transneuronal degeneration). These processes effectively impair the possibility for potentially regenerating axons to restore functionally useful connections and for replacement of lost connections by circuit rewiring [143]. Dorsal root avulsion provides a clinically relevant condition for exploring mechanisms underlying anterograde transneuronal degeneration in particular and means to counteract this process.

## 7. Bridges for Dorsal Root Injury Repair

Cell-based bridges, acellular bridges, and their combinations are attractive strategies for the repair of the spinal cord and dorsal root injury. The overall purpose of these strategies is to promote functional recovery by (i) survival/growth support for intrinsic cells,(ii) disease modification to facilitate endogenous repair, and (iii) replacement of lost cells or introduction of relay cells. The implementation of these strategies for spinal cord injury is discussed by Guo [144]. Lin et al. analyze how stem cells and biomimetic material for spinal cord repair interact with the human immune system [145], and Rodríguez-Barrera addresses the potential of recruitment from endogenous stem cells of cells for spinal cord injury repair [146]. Here, we discuss bridges aimed to promote functional growth of injured dorsal root axons into the spinal cord.

### 7.1. Cell-Based Bridges

Early studies aimed to provide a bridge for regenerating dorsal root axons into the spinal cord by exploiting, for example, Schwann cells [147], or activated macrophages [148], both types of cells which cooperate in peripheral nerve regeneration. These attempts were not successful, however; Schwann cells do not readily integrate into a CNS environment [32,149,150], and activated macrophages in a CNS environment tend to induce axonal retraction rather than extension [151].

**Figure 3:**
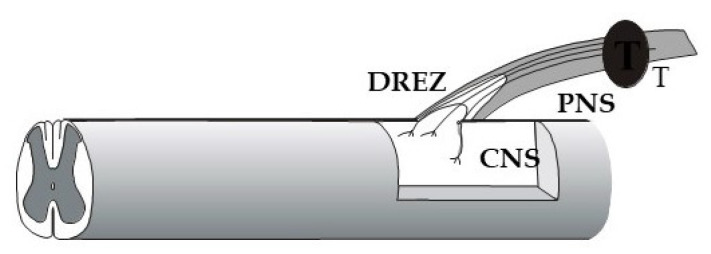
Scheme of experiment with human embryonic/fetal dorsal root ganglion (DRG) transplant (T) in the cavity of the extirpated native DRG of an adult rat recipient. Sensory axons from the transplant grow through the host DREZ and make synaptic contacts in the host spinal cord. For further information. Reproduced with permission from Kozlova, E.N., Seiger, A., Aldskogius, H., Journal of Neurocytology; published by Springer, 1997 [152,153].

With the purpose of probing the greater capacity for immature neurons to regenerate after injury, human embryonic/fetal dorsal root ganglion cells were implanted into the cavity of extirpated rodent DRGs (Figure 3). Sensory axons from the implant entered the host spinal cord along blood vessels, made functional synaptic connections there, and projected axons into the peripheral nerve [152,153]. These findings provided proof of principle that the barrier between the peripheral and central nervous systems can be overcome in adulthood. This sensory conduit from the periphery to the spinal cord circuitry was possible to create with highly growth competent sensory neurons, even without interfering with the non-permissive DREZ environment, through their ability to circumvent the glial barrier by extending along blood vessels [152].

Olfactory ensheathing cells (OECs) are CNS glia, ensheathing non-myelinated olfactory nerve axons in the intact nervous system, and able to serve as a cellular substrate for regenerating axons. OEC implants readily integrate into non-olfactory CNS regions and have been shown to be able to provide a pathway for sensory axon growth into the spinal cord and restore sensorimotor function after spinal root avulsion [154].

**Figure 4:**
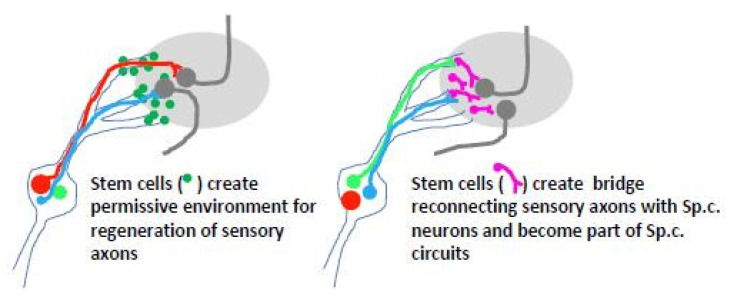
Two options based on the use of stem cell-based transplants at the DREZ, to facilitate entry of injured dorsal root axons (**left**) or to create a neuronal relay between dorsal root axons (forming synapses with the relay) and the host spinal cord circuitry (**right**).

Stem cell-based transplants at the DREZ could serve as facilitators for entry of dorsal root axons or as neuronal relays to interconnect dorsal root axons in the PNS compartment with spinal cord neurons (Figure 4). Since boundary cap cells allow sensory axons to enter the spinal cord during development (see Section 2), stem cells generated in vitro from this location were transplanted to the site of dorsal root avulsion in the mouse [155,156]. Transplanted cells survived, formed cellular bridges in the peripheral dorsal root compartment, partially migrated into the spinal cord, and underwent diverse neural differentiation but failed to support axonal ingrowth into the spinal cord. A possible explanation for this failure is that transplanted boundary cap neural crest stem cells contribute to a population of cells that express the growth repelling protein Mts1/S100A4 [114].

Human neural progenitor cell implants were shown to support sensory axon ingrowth and partial recovery of sensory function after dorsal root avulsion, seemingly by creating channels, i.e., acting as growth facilitators [157]. When such transplants were combined with nanoparticle-mediated delivery of growth factor peptide mimetics to the DREZ, sensory axon ingrowth was aborted [64]. Further studies indicated that growth factor administration accelerated progenitor cell maturation, probably causing a decline in their growth supportive properties [64]. This finding indicates that a combinatorial treatment strategy does not necessarily provide the anticipated additive or synergistic effects. However, combining platelet-rich plasma gel, a likely source of multiple growth factors, and human embryonic stem cell transplants allowed sensory axon ingrowth into the spinal cord [158].

In addition, to provide growth-supporting substrate, OEC or stem cell implants are likely to release growth supportive agents and presumably modify immune responses in a way that does not antagonize sensory axon growth across the DREZ. Clarifying the mechanisms underlying these approaches and explore the way to improve their efficiency is thus likely to contribute to the exploitation of cell-based therapies in direct spinal cord injury, e.g., by the administration of exosomes containing defined growth supporting and/or disease-modifying components [144,159].

### 7.2. The Dorsal Root as Pathway for Bridging Spinal Cord Injury

Pioneering studies by Aguayo and collaborators demonstrated that injured CNS neurons are able to elongate for long distances within a peripheral nerve graft [160,161,162]. As an extension of these findings, it became clear that lack of a growth-supporting environment is a key factor underlying the regeneration failure of injured axons in the CNS. In line with this, injured CNS axons fail to leave a peripheral nerve graft and re-enter the CNS and make functional connections due to the establishment of a non-permissive environment at the interface between the graft and the CNS tissue [163].

**Figure 5:**
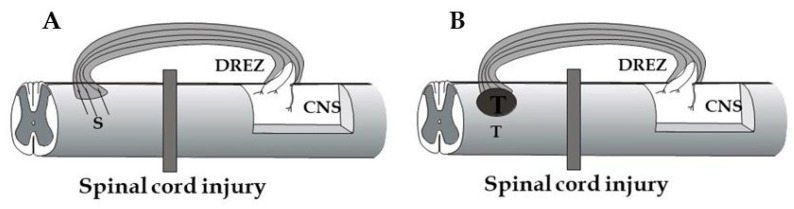
Principle scheme to use the DREZ as an entry point for axons growing in a bridge circumventing a spinal cord injury. (**A**) Axons from injured descending pathways (S) grow into and within a peripheral nerve graft (or biomimetic conduit) and may be able to enter the spinal cord through the intact or drug/cell modified DREZ. (**B**) Axons from an intraspinal transplant (T) grow through the bridge and may, because of their immaturity, be able to grow through the intact or reactive DREZ.

The application of bridges that circumvent a severely damaged spinal cord area would be a relevant option for restoring descending control of specific spinal cord functions. To make the axons elongating in the bridge enter the spinal cord, the graft-cord barrier issue needs to be resolved. This situation has similarities to the growth repelling barrier facing injured dorsal root axons at the DREZ. Approaches, which are discussed above to promote dorsal root regeneration into the spinal cord, are thus likely to be relevant also for achieving entry of axons, which have grown through a bridging graft. One additional, interesting aspect in this context is that the intact DREZ does not appear to exhibit a growth repellent barrier [164]. Figure 5 shows two possible approaches exploiting the modified or intact DREZ as an entry site for bridging a spinal cord injury, either by host axons growing through a peripheral nerve or biomimetic graft (A) or by axons from an intraspinal, stem cell-derived neuronal transplant (B).

### 7.3. Bioprinting and Dorsal Root Injury Repair

3D bioprinting has rapidly developed as a highly attractive technology for biomimetic implants that is able to support neuronal survival, counteract disease driving processes and support functional repair following spinal cord injury [144]. Based on detailed information on the underlying pathophysiological processes and conditions of the patient, 3D bioprinted implants may be designed to match patient-specific conditions [165]. Recent studies have demonstrated 3D bioprinted constructs that support neurite outgrowth from dorsal root ganglion neurons in vitro [166,167,168]. Dorsal root injury would be a useful in vivo model for exploring these developments and their further translation to the injured spinal cord.

## 8. Conclusions

Injuries that interrupt dorsal root axons result in loss of sensorimotor functions and often neuropathic pain, symptoms which are usually most serious following root avulsion injury. Injured dorsal root axons are unable to restore lost connections due to insufficient regeneration performance in combination with the encounter of a growth repelling CNS environment, i.e., factors which are closely similar to those that underlie the regeneration failure after direct spinal cord injury. Dorsal root injury offers attractive experimental models to determine key mechanisms in these processes in vivo, their exploitation for the development of novel therapeutic strategies, and their translation to a clinical setting for dorsal root injuries.

Dorsal root avulsion, which constitutes a longitudinal spinal cord injury, is of particular relevance in the broader context of spinal cord injury A successful strategy to tackle dorsal root avulsion injury most likely requires a combinatorial approach, applied in an appropriate spatial and temporal manner. We envision such a strategy might include the following actions. (i) Cell-based approaches, e.g., via exosomes derived from primed stem cells or as bioprinted constructs, may be suitable for promoting tissue repair at the injury site., as well as for creating pathways across the DREZ. (ii) Intrathecal administration of agents that specifically counteract non-neuronal axon growth inhibitors at the DREZ, as well as along ascending sensory pathways, are likely to be needed as additive measures. (iii) Application of gene regulatory tools to initiate and achieve sustained axonal growth by all subpopulations of dorsal root ganglion neurons. The dorsal root-spinal cord unit provides a favorable platform for assessing the outcome of these interventions, individually as well as in various combinations, initially in small rodent models and subsequently translated to larger species prior to clinical trials in humans.

## Data Availability

The authors’ findings and data referred to in the article are available from the corresponding author on reasonable request.
